# Supplementary material for: Variation of metabolic and bariatric surgery utilization by neighborhood socioeconomic status in Maryland
Source: PLoS One. 2025 Nov 10;20(11):e0330318. doi: 10.1371/journal.pone.0330318 (PMC12599945; doi:10.1371/journal.pone.0330318)
Supplement: Attachment — Submitted filename: Response to Reviewers_10202025.docx [file pone.0330318.s002.docx]

**Dear Editor and Reviewers,**

Thank you for your thoughtful evaluation of our manuscript and for the opportunity to revise it. We appreciate the insightful comments from the Academic Editor and both reviewers, and we have carefully addressed each point. Below, we provide a point‑by‑point response and a summary of changes made to the revised manuscript.

**Editor’s Comments**

1. **Use of IFSO‑compliant nomenclature.** We have replaced the term “bariatric surgery” with “metabolic and bariatric surgery (MBS)” throughout the manuscript, including in the title, abstract, tables, and narrative text. The abbreviation MBS is now defined at first use and used consistently thereafter.
2. **STROBE checklist.** A completed STROBE checklist is provided as a separate file. In it, we indicate the page and section in the manuscript where each reporting item is addressed.
3. **Causal language.** We revised the manuscript to avoid causal or policy‑prescriptive wording, reflecting the observational and cross‑sectional nature of our study. Statements that previously implied causality (for example, “effect of nSES on MBS use”) now describe associations (e.g., “association between nSES and MBS utilization”), and we emphasized that causal inferences cannot be drawn from these data.
4. **Limitations.** The limitations section has been expanded and now more clearly articulates how unmeasured confounding, selection bias, measurement limitations, and the cross‑sectional design may influence interpretation of our findings. We explicitly note that age and insurance distributions vary across socioeconomic strata and may confound the observed associations, and that our reliance on administrative data may lead to misclassification of comorbidities and procedures (Page 16-17, Lines 332-360).

In addition, we have updated the Data Availability Statement and checked the reference list for completeness and currency, replacing any outdated citations.

**Reviewer #1**

1. **Use “Metabolic and Bariatric Surgery (MBS)” instead of “bariatric surgery”.** As noted above, the nomenclature has been updated throughout the manuscript, including the title and table captions.
2. **Expand discussion of referral bias, racism, and hospital distance.** The Discussion now includes a paragraph addressing potential referral bias and structural racism in access to surgical care, citing relevant literature. We also discuss how geographical distance to MBS‑capable hospitals may reduce utilization among individuals in distressed neighborhoods (Discussion, Page 15, Lines 293-306).
3. **Insurance as mediator or confounder.** We added text to the Discussion noting that insurance coverage may function as both a mediator and a confounder in the relationship between neighborhood socioeconomic status and MBS utilization. We caution that Medicaid and uninsured status can both reflect socioeconomic disadvantage and limit access to surgery, and we note that further causal mediation analyses are beyond the scope of our cross‑sectional study (Page 15, Lines 307-323).
4. **Description of Figure 1.** The Results section now briefly describes Figure 1, highlighting the geographic distribution of neighborhood distress across Maryland and the corresponding variation in MBS utilization (Page 13, Lines 257-259).

**Reviewer #2**

We appreciate the reviewer’s observation that age and insurance coverage may confound our results. To address this, we conducted additional analyses of age, insurance and other study variables distributions across DCI quintiles and present these results in a new Table 2 (Pages 10-11, Lines 193-209, Page 26-27). The table shows that distressed neighborhoods have a higher proportion of younger adults (27.0 % aged 18–44 years vs. 20.9 % in prosperous areas) and a higher prevalence of Medicaid coverage (31.6 % vs. 11.9 %), whereas private insurance is more common in prosperous communities (41.1 % vs. 20.6 %). These differences are summarized in the Results (Page 10, Lines 193-209) and discussed in the context of potential confounding in the Discussion (Page 15, Lines 307-324).

We thank the reviewers again for their constructive feedback. We believe that these revisions have strengthened the manuscript and clarified the interpretation of our findings. We hope the revised submission meets the standards of *PLOS ONE* and look forward to your decision.

Sincerely,

Oluwasegun Akinyemi, MD, MSc., PhD (on behalf of all authors)
